# A Novel Fiber Optic Based Surveillance System for Prevention of Pipeline Integrity Threats

**DOI:** 10.3390/s17020355

**Published:** 2017-02-12

**Authors:** Javier Tejedor, Javier Macias-Guarasa, Hugo F. Martins, Daniel Piote, Juan Pastor-Graells, Sonia Martin-Lopez, Pedro Corredera, Miguel Gonzalez-Herraez

**Affiliations:** 1 FOCUS S.L., 28804 Madrid, Spain; javier.tejedor@focustech.eu (J.T.); hugo.martins@focustech.eu (H.F.M.); daniel.piote@focustech.eu (D.P.); 2 Department of Electronics, University of Alcalá, 28801 Alcalá de Henares, Spain; juan.pastor@depeca.uah.es (J.P.-G.); sonia.martin@depeca.uah.es (S.M.-L.); miguelg@depeca.uah.es (M.G.-H.); 3 Instituto de Óptica, CSIC, 28006 Madrid, Spain; p.corredera@csic.es

**Keywords:** distributed acoustic sensing, fiber optic systems, *ϕ*-OTDR, pipeline integrity threat monitoring, feature-level contextual information, system combination

## Abstract

This paper presents a novel surveillance system aimed at the detection and classification of threats in the vicinity of a long gas pipeline. The sensing system is based on phase-sensitive optical time domain reflectometry (*ϕ*-OTDR) technology for signal acquisition and pattern recognition strategies for threat identification. The proposal incorporates contextual information at the feature level and applies a system combination strategy for pattern classification. The contextual information at the feature level is based on the tandem approach (using feature representations produced by discriminatively-trained multi-layer perceptrons) by employing feature vectors that spread different temporal contexts. The system combination strategy is based on a posterior combination of likelihoods computed from different pattern classification processes. The system operates in two different modes: (1) machine + activity identification, which recognizes the activity being carried out by a certain machine, and (2) threat detection, aimed at detecting threats no matter what the real activity being conducted is. In comparison with a previous system based on the same rigorous experimental setup, the results show that the system combination from the contextual feature information improves the results for each individual class in both operational modes, as well as the overall classification accuracy, with statistically-significant improvements.

## 1. Introduction

Fiber optic distributed acoustic sensing (DAS) with phase-sensitive optical time-domain reflectometer (*ϕ*-OTDR) technology has been shown good performance for long perimeter monitorization aiming at detecting intruders on the ground [1,2,3,4,5] or vibration in general [6,7,8,9,10,11,12,13,14]. Current pipeline integrity prevention systems combine DAS technology and pattern recognition systems (PRS) for continuous monitoring of potential threats to the pipeline integrity [15,16,17,18,19,20,21,22].

In a previous work [22], we presented the first published report on a pipeline integrity threat detection and identification system that employs DAS + PRS technology, which was evaluated on realistic field data and whose results are based on a rigorous experimental setup and an objective evaluation procedure with standard and clearly-defined metrics (the original system was developed under a GERG (The European Gas Research Group)-supported project titled PIT-STOP (Early Detection of Pipeline Integrity Threats using a SmarT Fiber-OPtic Surveillance System)). In [22], we did a thorough revision of all of the previous published works in this area, showing their main limitations related to the pattern classification design: classification results were not presented; there was a lack of rigorous and realistic experimental conditions (database building, signal acquisition in limited distances); or they were aimed at a small number of classes (see [22] for more details).

More recently, new works on this topic have been published: In [19], there is again a lack of realistic experimental conditions since all of the signals corresponding to the same event are recorded in the same fiber position (hence, biasing the system to recognize the position instead of the real event); the sensed area covers up to 20 km (which reduces its application in realistic fiber deployments); and only five classes are employed. In [21], the sensing area spreads 24 km, and the real experiments were conducted at a fixed distance of 13 km away from the sensor (which we demonstrated in [22] was a major issue when facing realistic environments), dealing with only three classes. In addition, the number of tested signals in both works is small, with no additional details regarding the actual recording durations. Therefore, we can say that, again, these new systems do not fully address a realistic experimental setup that can assess the suitability of their proposals for realistic real-time monitoring of long pipelines.

The database used for the experiments in our previous work [22], which is composed of more than 1700 acoustic signals (about 10 h of recordings), addresses all of these issues: different events were recorded and tested in different positions (covering different soil conditions) and different days (covering different environmental conditions) along a 40-km pipeline. This, along with the adoption of a rigorous experimental procedure, allow us to state that the results are realistic enough to consider that similar performance can be obtained in field conditions.

With respect to the pattern recognition systems, one of the successful strategies used to improve their performance rates is adding contextual information [23]. For example, speech recognition systems obtain significant performance gains by incorporating context-dependent acoustic model information [24,25], or augmented features extracted from consecutive feature vectors (so-called first- and second-order derivatives [26]). Image recognition systems also obtain significant improvements by incorporating contextual information within the final classification rule from multiple objects that appear in the image [27].

In the field of fiber optic sensing, contextual information has also been employed for temperature measurement [28,29]. Our previous work [22] addressed the contextual information in a limited extent, since the short-time fast Fourier transform (ST-FFT) employed in the feature extraction spreads only one second (this was the optimal window size after an intensive experimentation with shorter and longer window sizes for the ST-FFT, all of them leading to lower system performance). Wavelets have also been employed previously to detect vibrations in distributed acoustic sensing systems, hence addressing contextual information to some extent, as well [30]. Both approaches show a strategy based on adding sample-level contextual information, which means that the original signal is processed taking into account each sample context. However, the contextual information is usually applied within pattern classification systems at the feature level [31,32,33,34], once the high dimensionality present in the input signal is reduced to a more discriminative set of features, which is more relevant for classification.

Another successful strategy to improve the performance of pattern recognition systems relies on system combination. This is based on the fact that complementary errors are provided by different pattern classification processes. The combination based on sum, product, average or maximum rules [35,36,37], majority voting [35,37] or more advanced techniques, such as logistic regression [38], Dempster-Shafer theory of evidence [37] and neural networks [36,37,39], have been applied to pattern recognition systems in different fields such as image recognition, speaker verification, handwritten recognition and speech recognition, showing significant performance gains.

### Motivation and Organization of the Paper

The pipeline integrity threat detection and identification system presented in previous works [15,16,17,18,19,20,21,22,40,41] did not make use of feature-level contextual information, nor did it exploit the possibility of combining results from different pattern recognition systems. Given the potential of both strategies, we propose to apply them on DAS + PRS technology for pipeline integrity threat detection and identification from two different perspectives:

- Incorporating feature-level contextual information in an intelligent way, adapting the so-called tandem approach widely used in speech recognition [42] to enhance the feature vector of the baseline system.
- Combining the outputs of different pattern classification processes, each of them using a combination of frequency-based and tandem features, exploiting different temporal ranges of contextual information.

In this paper, we present (to the best of our knowledge) the first published report that incorporates contextual information at the feature level and system combination in a DAS + PRS-based pipeline integrity threat detection and identification system, rigorously evaluated on realistic field data, showing significant and consistent improvements over our previous work [22].

The rest of the paper is organized as follows: The baseline system is briefly reviewed in Section 2, and Section 3 describes the novel pipeline integrity threat detection system. The experimental procedure is presented in Section 4, and the experimental results are discussed in Section 5. Finally, the conclusions are drawn in Section 6 along with some lines for future work.

## 2. Baseline System

### 2.1. Sensing System

The DAS system we used is a commercially available *ϕ*-OTDR-based sensor (named FINDAS) manufactured and distributed by FOCUS S.L. (Madrid, Spain) [43].

For interested readers, a full theoretical revision of the sensing principle and a detailed description of the experimental setup used in the FINDAS sensor can be found in [44], but we provide here a short summary of the sensing strategy used. The *ϕ*-OTDR makes use of Rayleigh scattering, an elastic scattering (with no frequency shift) of light, which originates from density fluctuations in the medium, to measure changes in the state of a fiber. In the FINDAS sensor employed, highly coherent optical pulses with a central wavelength near 1550 nm are injected into the optical fiber. The back-reflected signal from the fiber is then recorded, so that the interference pattern resultant from Rayleigh backscattering (*ϕ*-OTDR signal) is monitored at the same fiber input. By mapping the flight time of the light in the fiber, the *ϕ*-OTDR signal received at a certain time is associated with a fiber position. If vibrations occur at a certain position of the fiber, the relative positions of the Rayleigh scattering centers will be altered, and the *ϕ*-OTDR signal will be locally changed, thus allowing for distributed acoustic sensing [44].

The FINDAS has an (optical) spatial resolution of five meters (readout resolution of one meter) and a typical sensing range of up to 45 km, using standard single-mode fiber (SMF). A sampling frequency of $f_{s}=1085$ Hz was used for signal acquisition. A detailed description of the FINDAS technology can be found in [44].

### 2.2. Pattern Recognition System

The baseline PRS was based on Gaussian mixture models (GMMs) and conducted classification in two different modes:

1. The machine + activity identification mode identifies the machine and the activity that the machine is conducting along the pipeline.
2. The threat detection mode directly identifies if the activity is an actual threat for the pipeline or not.

**Figure 1:**
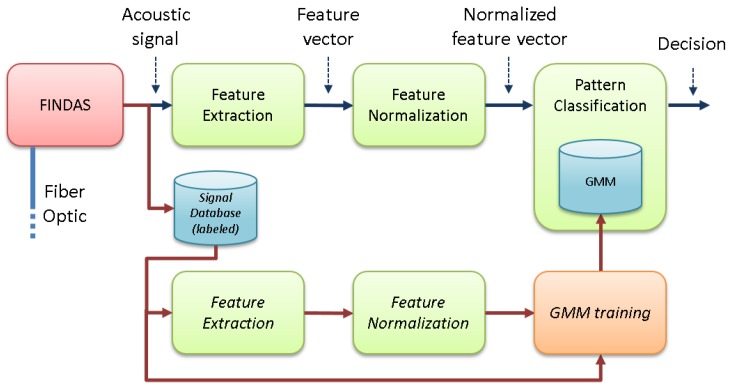
Baseline version of the system architecture [22].

The whole system integrated three main stages, as shown in Figure 1:

- Feature extraction, which reduces the high-dimensionality of the signals acquired with the DAS system to a more informative and discriminative set of features.
- Feature vector normalization, which compensates for variabilities in the signal acquisition process and the sensed locations.
- Pattern classification, which classifies the acoustic signal into a set of predefined $N_{C}$ classes (using a set of signal models, GMMs, previously trained from a labeled signal database).

This system obtained promising results taking into account the ambitious experimental setup (i.e., recordings in a real industrial deployment). However, the absolute performance rate in machine + activity classification (45.15%, far better than the 12.5% chance rate for $N_{C}=8$ classes) is still not high enough for a practical system in field operations. Even though the threat/non-threat classification rates were much better (80% of threat detection and 40% of false alarms), strategies to improve both rates are necessary.

The initial performance target that the GERG partners fixed to consider the system deployment in the field was over 80% for the threat detection rate and below 50% for the false alarm rate, so that these targets are actually achieved by the current proposal. With respect to the performance target for the machine + activity identification rates, the GERG partners did not impose any specific requirements, as the crucial aspect for real-world deployment is accurate threat detection. Considering the difficulty of the task (with eight different classes), identification rates in the range of 70%–80% are reasonable to start with.

## 3. Novel Pipeline Integrity Threat Detection System

**Figure 2:**
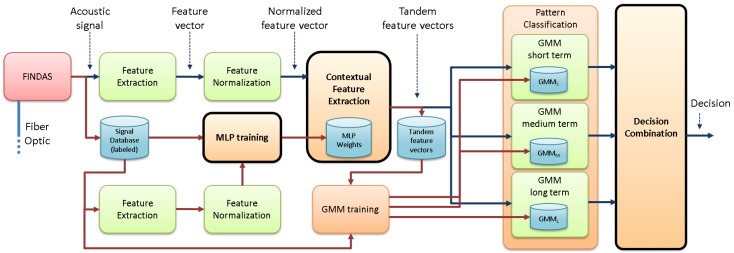
Novel pipeline integrity threat detection system architecture. Modules in bold typeface are the new ones with respect to [22].

The proposal of the novel pipeline integrity threat detection system is presented in Figure 2. First, the input acoustic signal is sent to a feature extraction module, where the energy corresponding to *P* frequency bands is calculated for the considered bandwidth $f\in [f_{0},f_{BW}]$, with $f_{0}$ and $f_{BW}$ being the initial and final frequencies respectively, and $f_{BW}\leq \frac{f_{s}}{2}$. This builds $N_{P}-\mathrm{dimensional}$ feature vectors ($N_{P}=100$). The feature normalization employed in this work is the sensitivity-based normalization described in Section III.B.2 of [22], where each coefficient of those feature vectors is normalized by the energy above the considered bandwidth. This was necessary due to the strong differences in the signals acquired in different sensing positions, which relate to the different soil conditions, the mechanical coupling of the fiber to the pipe enclosure, the machinery distance, the non-linear transduction function of a *ϕ*-OTDR-based sensor, the exponential decay of the amplitude of the measured signals along the fiber, etc. (see [22] for more details). The pattern classification module employs a GMM-based approach to classify each feature vector into the most likely class (machine + activity pair in the machine + activity identification mode that deals with $N_{C}=8$ classes, and threat/non-threat in the threat detection mode that deals with $N_{C}=2$ classes). This employs the a posteriori maximum probability criterion to assign the given feature vector the class with the highest probability given by the corresponding GMM. The additional blocks, the contextual feature extraction (that also needs a new previous training stage) and the decision combination are new with respect to our previous work [22] and are explained in more detail next.

### 3.1. Contextual Feature Extraction

The contextual feature extraction is based on the tandem approach used to compute the so-called tandem features in speech recognition tasks [45,46,47]. This module takes the normalized frequency-based feature vectors as input and produces tandem feature vectors as output.

**Figure 3:**
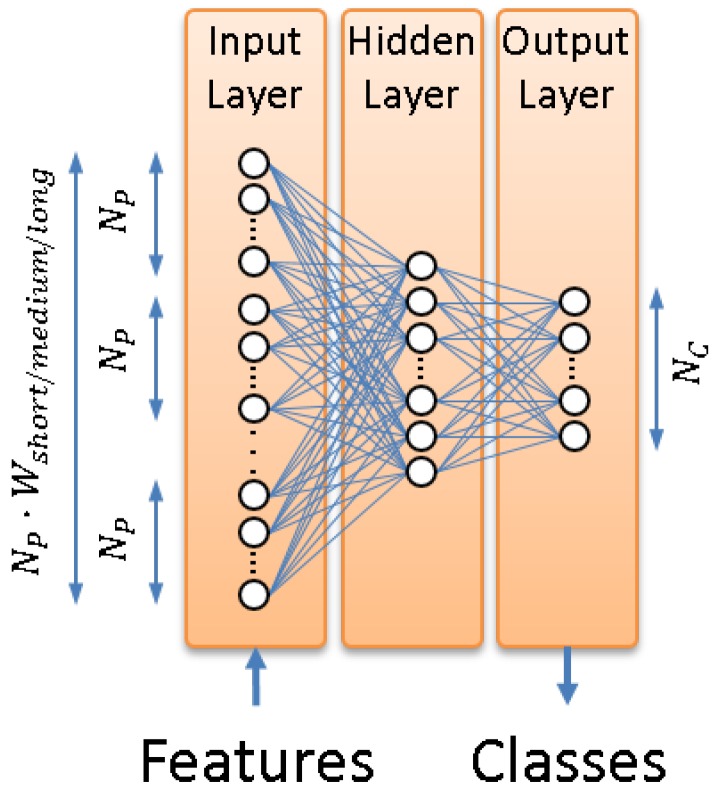
Architecture of the three-layer MLP employed in the contextual feature extraction module.

A multi-layer perceptron (MLP) is employed to integrate the feature-level contextual information. This MLP has three layers, as shown in Figure 3: an input layer that consists of $N_{P}\cdot W_{size}$ feature vector values, where $W_{size}$ is the number of feature vectors used as contextual information (for an acoustic frame being analyzed at time *t*, the MLP will use the $W_{size}/2$ feature vectors before *t* and the $W_{size}/2$ feature vectors after *t*, along with the feature vector generated for time *t*), a hidden layer, whose number of units is selected based on preliminary experiments, and an output layer, with the number of units equal to the number of classes involved in the system modes (eight in the machine + activity identification mode and two in the threat detection mode).

Specifically, three MLPs will be used to model the behavior of short, medium and long temporal contexts, using $W_{short}$, $W_{medium}$ and $W_{long}$ feature temporal window sizes, respectively. The objective is effectively dealing with different signal behaviors that cope with short, medium and long temporal contexts, so that a wider range of activities can be better learned by the system. In our implementation, the time lengths of each temporal context are 5 s, 12.5 s and 20 s, corresponding to the short, medium and long temporal contexts, respectively. These lengths were chosen based on the length of a single behavior within different activities. For example, for stable activities, such as moving, long temporal windows are more suitable to model a single behavior. However, for more difficult activities (hitting or scrapping that include several behaviors), shorter temporal windows are preferable so that the temporal windows used for modeling better cope with generating a robust model for a single behavior.

**Figure 4:**
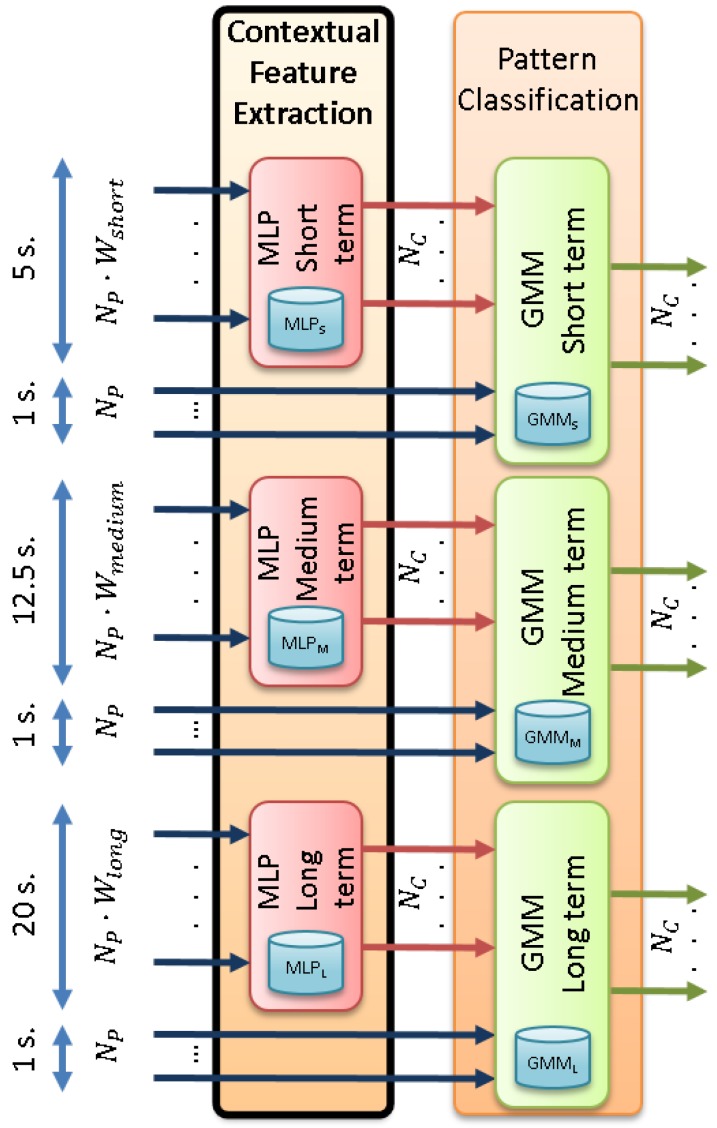
Detailed architecture of the contextual feature extraction module and its connection to the GMM-based pattern classification modules.

Figure 4 shows the detailed architecture of the contextual feature extraction module and its connection to the GMM-based pattern classification modules.

The MLP models required for each temporal context (referred to as ${\mathrm{MLP}}_{S}$, ${\mathrm{MLP}}_{M}$ and ${\mathrm{MLP}}_{L}$ in Figure 4) are trained by the MLP training module in Figure 2. The standard back-propagation algorithm [48] is employed to learn the MLP weights (i.e., connections between all of the units of the input and hidden layers and connections between all of the units of the hidden and output layers, as shown in Figure 3). Therefore, three different sets of weights are learned (one for each temporal context), which are used next to obtain the posterior probability vectors.

The contextual feature extraction involves two different stages, which are applied to each of the different temporal contexts:

#### 3.1.1. Posterior Probability Vector Computation

For each set of normalized feature vectors and using the weights computed during MLP training, the MLP is employed to calculate a posterior probability for each class to be identified. This process is similar to using the MLP for classification. However, instead of assigning a raw class label to each normalized feature vector, the MLP outputs (consisting of one posterior probability per class, as shown in Figure 3) are used as new features. This builds a set of $N_{C}$-dimensional posterior probability vectors per MLP (i.e., per temporal context), as shown in Figure 4.

#### 3.1.2. Tandem Feature Vector Building

This stage concatenates the original $N_{P}$-dimensional feature vectors (those generated by the feature normalization module) and the $N_{C}-\mathrm{dimensional}$ posterior probability vectors computed by the MLPs. Therefore, $(N_{P}+N_{C})-\mathrm{dimensional}$ tandem feature vectors are built (in our implementation, $N_{P}+N_{C}=108$ for the machine + activity identification mode and $N_{P}+N_{C}=102$ for the threat detection mode). These are fed into three different pattern classification processes (one for each temporal context), which generate a likelihood value for each of the $N_{C}$ classes, as shown in Figure 4. It must be noted that the GMM training is also carried out from these tandem feature vectors.

For MLP training, posterior probability vector computation and tandem feature vector building, the ICSI QuickNet toolkit [49] has been employed.

### 3.2. Decision Combination

Given the three pattern classification processes conducted on the tandem feature vectors that cover different temporal contexts and in order to exploit their complementarity when dealing with different activities, a way to combine their outputs is necessary. In this work, we have evaluated three methods to carry out a likelihood-based combination: sum, product and maximum, which are presented next:

#### 3.2.1. Sum Method

For any frame (i.e., feature vector), the likelihood assigned to each class $c_{i}$ is given by:

(1) $$l\left(c_{i}\right)=\sum_{j=1}^{N}l_{j}\left(c_{i}\right),$$

where *N* is the number of classification processes and $l_{j}\left(c_{i}\right)$ is the likelihood assigned to class $c_{i}$ in the classification process *j*.

This sum method is typically better adapted for cases in which each classifier performs different [50].

#### 3.2.2. Product Method

For any frame, the likelihood assigned to each class $c_{i}$ is given by:

(2) $$l\left(c_{i}\right)=\prod_{j=1}^{N}l_{j}\left(c_{i}\right).$$

This product method is typically better adapted for systems where the feature sets are independent [51].

#### 3.2.3. Maximum Method

For any frame, the likelihood assigned to each class $c_{i}$ is given by:

(3) $$l\left(c_{i}\right)=\max_{j=1}^{N}l_{j}\left(c_{i}\right).$$

This maximum method is typically better adapted for systems where the performance of each individual classifier is similar [50].

For all of the combination methods, the class that is finally assigned to each frame as the recognized one is given by the maximum a posteriori criterion:

(4) $$\hat{c}=\underset{i}{argmax}\left\{l(c_{i})\right\}.$$

The combination approach can be applied to all of the classification processes, or to a selection of them, so that a fruitful experimentation can be carried out.

## 4. Experimental Procedure

Our experimental setup is basically the same as that described in Section IV of [22]. We provide here the fundamental details, referring the reader to the original paper for further details.

### 4.1. Database Description

For comparison purposes, we employed the same database as in our previous work [22], whose content is summarized in Table 1.

As described in [22], an active gas transmission pipeline operated by Fluxys Belgium S.A. was used for the database acquisition, thus operating in a real scenario. The pipeline is made from steel, has a diameter of one meter and is one inch thick. Activities nearby the pipeline were sensed by monitoring an optical fiber cable installed about 0.5 m from the pipeline and parallel to it (the fiber cable installation was done at the same time of the pipeline construction). The pipeline and the associated optical fiber are buried, and the pipeline is pressurized at 100 bars (being an active one, operating in normal conditions). The fiber depth varies between 0.3 and two meters, and since it does not follow a tight parallel path along the pipeline and in some points, there are fiber rolls for maintenance purposes, a calibration procedure between fiber distance and geographical location was carried out for precise location labeling.

The selected activities cover realistic situations (involving possible threats and harmless ones) that could typically occur nearby pipeline locations. All of them were carefully selected by the GERG partners within the PIT-STOP project and represented those activities that could provide the best assessment of the system capabilities for real-world deployment. In particular, the staff at Fluxys Belgium S.A. (the gas carrier company in this country) was responsible for the proposal of the activities to be carried out for evaluation.

On the one hand, the dangerous activities (hitting and scrapping by small and big excavators) allowed the system to be tested when a real threat for the pipeline occurs (as is the usual situation before a critical pipeline “touch” happens).

On the other hand, the non-threat activities were chosen based on their high occurrence rate near pipelines (movements of different machinery and non-dangerous activities performed by pneumatic hammer and plate compactor machines).

The FINDAS sensor is connected at one end of the fiber that runs in parallel to the inspected pipeline. The different locations (LOC1, LOC2, LOC3, LOC4, LOC5 and LOC6) cover different pipeline “reference positions” selected at high distances from the sensing equipment (being at 22.24, 22.49, 23.75, 27.43, 27.53 and 34.27 km far from the FINDAS box, respectively) to evaluate the system in conditions close to the actual sensing limits and to ensure feature variabilities in terms of soil characteristics and weather conditions (see [22] for more details).

**Figure 5:**
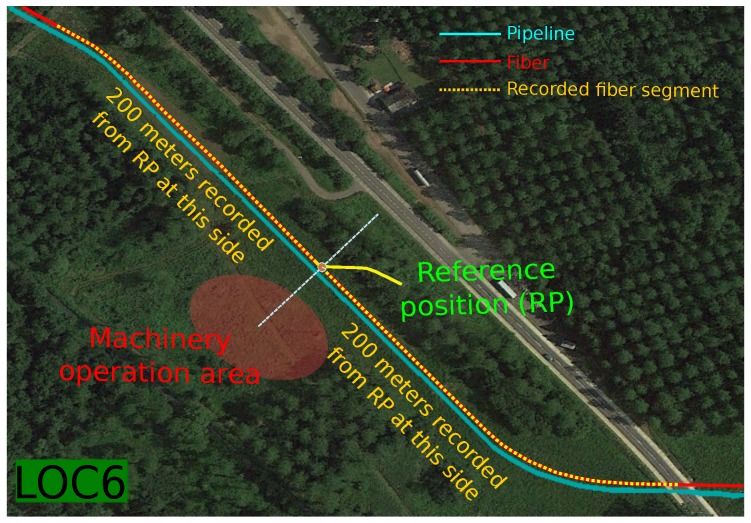
Recording scenario: real example at LOC6, taken from [22].

The machines used for the recordings of the different machine + activity pairs started their activity at the center of the so-called “machine operation area” (see Figure 5 for a visual reference). This area was located at distances between zero meters (on top of the fiber) and up to 50 m from the so-called “reference position” right above the pipeline (as described in [22] in the recording protocol for each location, the reference position was chosen manually as the closest to the center of the operation area with good sensitivity, by real-time monitoring of the fiber response). The “hitting” and “scrapping” activities were recorded five times in different positions within the machine operation area (the first position was located in the center of the area, and the other four were located at ±25 m and ±50 m from this center, with the direction depending on the available space around the operation area). The “movement” and “compacting” activities spread around ±25 m from the center of the operation area. These two activities were recorded in two different ways: the first one comprises both movement and compacting actions when the machine is carrying out the activity parallel to the pipeline, the second one with the activity carried out perpendicular to the pipeline. This allowed us to generate different acoustic patterns corresponding to both ways, hence obtaining a more varied database. From this “reference position”, the signals were captured from the optical fiber in a ±200-m interval (see Figure 5), with one-meter spacing, thus generating 400 acoustic traces for each recorded activity. This 400-m interval was selected to ensure that we had a wide enough range of fiber responses to be used in the training and evaluation procedures.

Although the distance of the acoustic source (the machine performing the given activity) to the optical fiber has an impact on the signal-to-noise ratio (SNR), the high sensitivity of the sensing system within the limits of the selected “machine operation area” for each location makes the SNR good enough to cover realistic and practical situations. Moreover, the trained signal models are also able to cope with this variability due to the acoustic source distance to the pipeline.

### 4.2. System Configuration

Regarding the feature extraction, the relevant parameters are as follows: The acoustic frame size was set to one second; the acoustic frame shift was set to five milliseconds; the number of FFT points was set to 8192; the number of frequency bands (i.e., the original feature vector size) was set to 100; and the initial and final frequencies corresponding to the analyzed bandwidth were set to 1 Hz and 100 Hz, respectively.

The highest energy meter selection in our previous work has been selected for signal representation, due to its better performance over the reference position (see Figure 5) [22]. Therefore, each acoustic frame used either for training or evaluation (MLP in the contextual feature extraction and GMM in the pattern classification) corresponds to the highest energy meter between those acquired by FINDAS.

For the contextual feature extraction, 100 units have been used in the hidden layer for MLP training and posterior probability vector computation for the machine + activity identification mode and three units for the threat detection mode. These values were chosen based on their best performance in preliminary experiments.

For pattern classification, a single GMM component has been used to model each class in both modes.

The use of the sensitivity-based normalization and the bandwidth limited to 100 Hz are explicitly designed to also help in dealing with the noise in the raw data. The normalization aids in equalizing noise effects compensating for variabilities in the signal acquisition process and the sensed location (as background noise can vary for different locations due to the proximity of road, factories, etc.), and the bandwidth limitation avoids considering noisy signals where no relevant information is to be found. Furthermore, while variations in the fiber temperature could introduce noise in the measurements, these typically occur at much lower frequencies than the processed acoustic signals, so that they do not constitute a relevant issue in our proposal. Nevertheless, even though the raw signals have a high level of noise (as shown in the sample signal spectrograms shown in Figure 2 of [22]), each machine + activity pair exhibits, in general, a reasonably consistent spectral behavior, hence allowing for the use of pattern classification strategies that can efficiently extract this consistent behavior. A full experimental and theoretical description of the optical noise characteristic of the DAS technology using a similar setup, which defines the background noise of the raw data, can be found in [44].

### 4.3. Evaluation Strategy

The evaluation strategy was carefully and rigorously designed to maximize the statistical significance of the results and to provide a wide variety in the design of the training and evaluation subsets.

With this objective, the robust and widely-adopted leave-one-out cross-validation (CV) strategy [52] was selected to carry out the experiments. The criteria to split the full database into training and evaluation subsets match with the recorded data location criteria. Since data were recorded in six different locations, the CV strategy comprises six folds, where the data recorded in all of the locations except one were used for training (including MLP training and posterior probability vector computation for the contextual feature extraction and GMM training for the pattern classification), and the evaluation was done on data of the unused location (thus ensuring full independence between the training and evaluation subsets). Classification is again conducted on a frame-by-frame basis.

Using the data from the same locations for MLP training and posterior probability vector computation in the contextual feature extraction could lead to overfitting problems, since a subset of the data employed for MLP training is also used to compute the posterior probability values of the tandem feature vectors employed for training the pattern classification module. To evaluate this drawback, we ran a full set of experiments in which different locations for MLP training and posterior probability vector computation were employed, and similar results are obtained, which clearly indicates that no overfitting occurs.

### 4.4. Evaluation Metrics

As in our previous work [22] and for comparison purposes, the classification accuracy has been the main metric to evaluate the system performance both for the machine + activity identification and threat detection modes. In addition, we will also show the class classification accuracy for the machine + activity identification mode and the threat detection rate and false alarm rate for the threat detection mode. Finally, to provide a full picture of the classification performance, we will also show the confusion matrix (i.e., a table that shows the percentage of evaluation frames of a given class that are classified as any of the considered classes) for the machine + activity identification mode. Statistical validation of the results will be provided to assess the statistical significance of the results.

## 5. Experimental Results

### 5.1. Preliminary Experiments

A preliminary set of experiments was run to show the potential effectiveness of (1) using contextual information and (2) combining different contextual information sources in the whole system.

This set of experiments takes the 100-dimensional normalized feature vectors as input for the MLP and conducts classification. For MLP-based classification, we simply assign the class with the highest posterior probability as the recognized class with which we can evaluate the system performance. The different temporal contexts (short, medium and long) are employed for MLP training and classification, and the obtained results are presented in Table 2.

From Table 2, it is clearly seen that, even though the overall accuracy improves when increasing the temporal context, the optimal temporal context (short, medium or long) is different for each machine + activity pair (the best rates are shown in bold). For example, for the big excavator moving, the baseline performance is 49.1%, and this increases to 63.3%, 72.9% and 82.5% when using progressively longer temporal contexts (short, medium and long, respectively). On the other hand, for the small excavator hitting, increasing the temporal context leads to systematic performance degradation from the 13.8% obtained in the baseline to 10.7%, 8.8% and 7.0% for progressively longer temporal contexts.

These results indicate that different temporal contexts model the feature space in a different way, so that employing and combining different window sizes could bring further improvements to the whole system performance (thus, motivating our combination approach). In addition, the MLP does not seem to be suitable to replace the GMM for classification. Despite the best overall performance obtained with the long-length window size, there are some classes whose performance is worse than that of the baseline (hitting and scrapping activities with the small excavator and hitting activity with the big excavator, which include multiple behaviors and have less training data). Therefore, this motivates the use of the MLP to produce a tandem feature vector and to maintain the GMM-based pattern classification system.

### 5.2. Contextual Feature Extraction

We analyze the performance of the contextual feature extraction module from the tandem feature vectors that are built from different window sizes. To do so, a GMM-based pattern classification process is carried out for each of the proposed temporal contexts (short, medium and long), as shown in Figure 4, and results are presented in Table 3.

At first sight, for the machine + activity identification mode, the average system performance compared with the baseline (column Acc. in Table 3) seems to improve to a great extent (57.8%−45.2%=12.6% absolute improvement). Paired *t*-tests [53] show that this improvement is statistically significant for any window size over the baseline ($p<10^{-32}$). However, looking at the individual class performance, this improvement is not that clear. There are classes for which very similar or even slightly worse performance is obtained with the tandem feature vectors (e.g., small excavator doing hitting (13.8% for the baseline system and 13.4% for the tandem system) and scrapping (30.2% for the baseline system and 30.3% for the tandem system)), and the best performance for each class largely depends on the window size.

The large improvement obtained with the tandem feature vectors is for the classes for which more data are available. For example, the moving activity from the big excavator improves the 49.1% baseline performance to 74.4% for the tandem system, and from the small , the improvement goes from the 50.5% baseline performance to 62.0%. Furthermore, large improvements are observed for the plate compactor (from 39.5% to 54.0%) and the pneumatic hammer (from 71.8% to 81.1%). The fact that more data are available for these classes is biasing the performance calculation, but we also have to consider the effect on the classes with lower performance. The high performance classes, which tend to have a more stable behavior, get much more benefit from the feature-level contextual information than classes that represent different acoustic behaviors (i.e., hitting and scrapping activities). The greater amount of training data of those classes also contributes to this, since a more robust GMM is trained.

On the contrary, for classes with different acoustic behaviors during the execution (hitting and scrapping), integrating these multiple behaviors could lead to less robust GMMs, so that the final performance for these classes is similar or even worse than that of the baseline. For example, for the small excavator hitting, there is a performance degradation from the baseline 13.8% to 13.4%. The only exception for this observation is the improvement obtained for the big excavator doing scrapping (36.9% versus 26.0% of the baseline), which may be due to the greater amount of training data available, so that a more robust GMM is built.

This suggests that using feature-level contextual information in isolation is not enough to obtain the best performance in the whole system for classes for which different acoustic behaviors are observed and the amount of data used to train the GMM is limited.

For the threat detection mode, it can be seen that incorporating feature-level contextual information also provides an improvement in the overall classification accuracy over the baseline (69.7%−64.3%=5.4% absolute improvement). Paired *t*-tests show that this improvement is statistically significant for any window size ($p<10^{-24}$) over the baseline. However, by inspecting the threat detection rate and the false alarm rate, it can be seen that both figures decrease compared with those of the baseline, which makes it more difficult to derive a clear conclusion.

From these results, we can state that decision combination is necessary to take advantage of the complementary classification errors obtained for each temporal context.

### 5.3. Decision Combination

Decision combination employs different combinations of temporal contexts (in pairs or all of them) to make the final decision for each frame. Results are shown in Table 4 for the machine + activity identification mode and the threat detection mode. To ease the analysis, the results for the sum method are not shown, as they are almost identical to those obtained with the product method. Additionally, the cells with worse results than the baseline have an orange background and the green background cells indicate the selected systems for the machine + activity identification and threat detection modes. As can be seen, almost all of the results obtained with the decision combination improve those of the baseline.

#### 5.3.1. Machine + Activity Identification Mode

For the machine + activity identification mode, the combination of any window size with any combination method outperforms the overall classification accuracy of the baseline to a great extent (52.91%−45.15%=7.76% minimum absolute improvement, which means a 17% relative improvement). Paired *t*-tests show that this improvement is statistically significant for all of the cases ($p<10^{-30}$).

For sum and product methods, consistent performance gains are obtained for all of the classes in general. The sum method is expected to work well when each individual classifier performs quite different [50], as is our case (see Table 3). The product method is also expected to derive a robust combination when the feature sets are independent [51]. Different temporal contexts model the feature space in a different way so that the feature set for every class can be considered as independent.

For hitting and scrapping activities, which possess multiple behaviors and have less training data, the performance obtained with the maximum method is much worse than that of the baseline (for example, for the small excavator hitting, the 13.8% baseline gets as low as 9.7%). This can be due to two reasons: (1) the maximum method does not integrate information of different classification processes (only the best likelihood is selected), which for multi-class classification problems is important, and (2) this method provides gains when the performance of the individual classifiers is close, which is not our case (see Table 3). The only exception is again for the big excavator doing scrapping, for which performance gains are obtained for each combination method (from the 26.0% baseline performance up to 36.3% with the product method and 36.9% with the maximum method). This may be again due to the availability of more training data, which results in a more robust GMM.

Our selection proposal is the product-based combination from medium and long temporal window sizes, since this presents the best overall accuracy with consistent improvements for each individual class.

Table 5 shows the corresponding confusion matrix of this combination, where we have removed the values below chance (1/8 = 12.5%) to ease the visualization and analysis and where we have used color information as a visual aid. In general, it is clearly seen that the diagonal contains the greatest figures for each class (with at least 9% absolute better accuracy compared to the second most recognized one, i.e., 33.74%−24.64% = 9.10% in the big excavator doing scrapping), except for the hitting activity. For the big excavator, this is confused with the moving and scrapping activities. On the one hand, the big excavator doing hitting has less training data, which can cause that the classification process prefers the GMM for which more training data are available. On the other hand, scrapping also includes hitting when the shovel contacts the ground, which is also causing confusion in the small excavator. The classes with the lowest performances correspond to the hitting and scrapping activities, which are also confused with each other. On the one hand, these are the classes with less training data, which derives a less robust GMM. In addition, hitting and scrapping activities present different acoustic behaviors (moving up the shovel, moving it down, hitting, scrapping, moving, etc.), which may degrade the GMM, since just a single GMM component is used for modeling (increasing the number of GMM components does not provide any gain, probably due to the small amount of training data for these classes).

It is also important to note the significant improvements in the identification rates with respect to the baseline system, as shown in Table 6. The relative performance improvement between the baseline and novel systems range from 4.48% up to 37.74%, with an average value of 21.30%, which clearly validates the strategy used towards improving the overall performance.

#### 5.3.2. Threat Detection Mode

For the threat detection mode, the overall classification accuracy shows a similar trend. All of the method combinations for any window size significantly outperform the baseline ($p<10^{-26}$ for a paired *t*-test).

Combining all of the temporal window sizes with the maximum method outperforms the baseline both for the threat detection rate (from the 80.7% baseline performance up to 81.1%, which implies a relative improvement of 0.5%) and the false alarm rate (from the 40.3% baseline performance down to 35.4%, which implies a relative improvement of 12%). These improvements are significant for the threat detection rate ($p<10^{-5}$) and for the false alarm rate ($p<10^{-28}$). By integrating all of the window sizes in a small classification task (two classes: threat/non-threat), the feature space is modeled in such a different way that the pattern classification makes different and complementary errors, so that the final performance gets improved in the maximum method, for which the classifier with the highest likelihood makes the final decision.

## 6. Conclusions and Future Work

This paper has presented a novel approach for a pipeline integrity threat detection system that employs a *ϕ*-OTDR fiber optic-based sensing system for data acquisition by adding feature-level contextual information and system combination in the pattern recognition stage. The proposal achieves consistent and significant improvements that were verified in a machine + activity identification task, where the machine and the activity carried out must be known, and in a threat detection task, where just the occurrence of a threat for the pipeline has to be known.

Feature-level contextual information in isolation has been shown to perform well for machine + activity pairs that possess a stable behavior and for which enough training data are available. Adding the decision combination from different pattern recognition processes that run on different contextual information window sizes has been shown to outperform the overall classification accuracy and the class classification accuracy for both tasks.

Although the results presented in this paper have improved those of the baseline to a great extent (about 21% relative to the machine + activity identification mode and 12% relative to the false alarm rate with a slight improvement of 0.5% relative to the threat detection rate for the threat detection mode), there is still much work to do. For classes for which different behaviors exist and the amount of training data is low, the improvements obtained are not as high as for the rest of the classes. Therefore, future work should focus on these low-performance classes by, for example developing new strategies that will also extend our system to make use of contextual information in the spatial domain (that is by using the acoustic traces from nearby sensed positions, which should experience similar disturbances simultaneously).

## Figures and Tables

**Table 1:** Experimental database. ”Big excavator” is a 5-ton Kubota KX161-3. ”Small excavator” is a 1.5-ton Kubota KX41-3V. From [22]. LOC, location.

Machine	Activity	Duration (in Seconds)							Threat/Non-Threat
		LOC1	LOC2	LOC3	LOC4	LOC5	LOC6	Total	
Big excavator	Moving along the ground	1100	1100	3540	1740	1620	4160	13,260	Non-threat
	Hitting the ground	120	140	240	220	80	260	1060	Threat
	Scrapping the ground	460	460	920	620	200	580	3240	Threat
Small excavator	Moving along the ground	600	500	1700	820	820	1660	6100	Non-threat
	Hitting the ground	200	180	220	220	80	240	1140	Threat
	Scrapping the ground	420	340	780	360	180	520	2600	Threat
Pneumatic hammer	Compacting ground	660	0	580	1320	0	1320	3880	Non-threat
Plate compactor	Compacting ground	740	0	740	1240	0	1680	4400	Non-threat

**Table 2:** MLP classification accuracy for the machine + activity identification mode for every class with various window sizes with the best result for each class in bold font. ”Acc.” is the overall classification accuracy, with the best result in bold font. ”Mov.” stands for moving; ”Hit.” stands for hitting; ”Scrap.” stands for scrapping; and ”Compact.” stands for compacting.

Window Size	Machine + Activity Identification								
	Big Excavator			Small Excavator			Pneumatic Hammer	Plate Compactor	Acc.
	Mov.	Hit.	Scrap.	Mov.	Hit.	Scrap.	Compact.	Compact.	
Baseline [22]	49.1%	20.1%	26.0%	50.5%	13.8%	30.2%	71.8%	39.5%	45.2%
Short	63.3%	13.0%	31.5%	54.8%	10.7%	26.5%	73.9%	57.3%	53.5%
Medium	72.9%	12.1%	35.4%	63.8%	8.8%	28.3%	76.9%	51.3%	58.6%
Long	82.5%	12.3%	34.5%	62.5%	7.0%	28.1%	82.2%	46.2%	61.8%

**Table 3:** Contextual feature extraction module results. Class classification accuracy and overall classification accuracy for the machine + activity identification mode and the threat detection rate (TDR), false alarm rate (FAR) and overall classification accuracy for the threat detection mode, with the best results in bold font. ”Acc.”, ”Mov.”, ”Hit.”, ”Scrap.” and ”Compact.” denote the same as in Table 2.

Window Size	Machine + Activity Identification									Threat Detection		
	Big Excavator			Small Excavator			Pneumatic Hammer	Plate Compactor	Acc.	TDR	FAR	Acc.
	Mov.	Hit.	Scrap.	Mov.	Hit.	Scrap.	Compact.	Compact.				
Baseline [22]	49.1%	20.1%	26.0%	50.5%	13.8%	30.2%	71.8%	39.5%	45.2%	80.7%	40.3%	64.3%
Short	60.6%	17.0%	32.0%	55.9%	11.6%	27.8%	75.6%	54.0%	52.8%	78.9%	36.3%	67.1%
Medium	66.1%	19.0%	36.9%	62.0%	10.8%	30.3%	75.9%	49.7%	56.0%	76.6%	32.3%	69.7%
Long	74.4%	21.5%	30.2%	59.2%	13.4%	28.5%	81.1%	43.4%	57.8%	71.6%	31.2%	69.4%

**Table 4:** Decision combination results. Class classification accuracy and overall classification accuracy for the machine + activity identification mode and the threat detection rate (TDR), false alarm rate (FAR) and overall classification accuracy for the threat detection mode with the best results in bold font. For combination, ”Prod” is the product method, and ”Max” is the maximum method. ”S” denotes short window size; ”M” denotes medium window size; and ”L” denotes long window size. ”Acc.”, ”Mov.”, ”Hit.”, ”Scrap.” and ”Compact.” denote the same as in Table 2.

Method		Machine + Activity Identification									Threat Detection		
		Big Excavator			Small Excavator			Pneumatic Hammer	Plate Compactor	Acc.	TDR	FAR	Acc.
		Mov.	Hit.	Scrap.	Mov.	Hit.	Scrap.	Compact.	Compact.				
Baseline [22]		49.1%	20.1%	26.0%	50.5%	13.8%	30.2%	71.8%	39.5%	45.15%	80.7%	40.3%	64.26%
**Prod**	S-M	59.9%	19.4%	36.3%	60.4%	13.0%	33.8%	75.8%	44.4%	53.06%	76.8%	33.2%	69.10%
	S-L	64.3%	23.7%	32.1%	57.7%	**18.0%**	31.1%	80.4%	40.1%	53.91%	74.9%	33.7%	68.25%
	M-L	66.1%	22.2%	33.7%	57.9%	14.3%	36.6%	78.4%	41.3%	54.92%	73.9%	**32.0%**	**69.32%**
	S-M-L	61.5%	**24.0%**	34.0%	57.6%	15.0%	**36.9%**	78.2%	39.8%	53.09%	75.0%	33.2%	68.68%
**Max**	S-M	67.3%	17.3%	**36.9%**	64.2%	9.7%	27.2%	79.5%	**56.6%**	57.75%	81.0%	36.2%	67.66%
	S-L	76.8%	17.2%	32.1%	62.9%	10.9%	29.4%	81.1%	50.0%	60.20%	79.7%	35.0%	68.29%
	M-L	76.6%	14.8%	34.2%	64.1%	11.5%	29.2%	80.1%	49.9%	60.33%	78.4%	33.4%	69.24%
	S-M-L	**77.0%**	14.5%	34.0%	**65.0%**	10.0%	27.8%	**81.7%**	51.4%	**60.82%**	**81.1%**	35.4%	68.34%

**Table 5:** Confusion matrix of the product combination method from medium and long window sizes for the machine + activity identification mode. Classification accuracy is shown in each cell. The values between brackets represent the number of frames that are classified as the recognized class or that belong to the real class.

				**Recognized Class**								
				**Big Excavator**			**Small Excavator**			**Pneumatic** **Hammer**	**Plate** **Compactor**	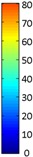
				[236845] **Moving**	[40432] **Hitting**	[81899] **Scrapping**	[94597] **Moving**	[61857] **Hitting**	[91389] **Scrapping**	[77049] **Compacting**	[56292] **Compacting**	
**Real class**	Big excavator	[275145]	Moving	**66.09**								
		[21995]	Hitting	**30.60**	**22.15**	**19.21**						
		[67230]	Scrapping	**24.64**		**33.74**			**18.39**			
	Small excavator	[126575]	Moving				**57.91**		**16.92**			
		[23655]	Hitting	**17.03**		**14.01**		**14.32**	**29.55**			
		[53950]	Scrapping			**15.55**		**12.62**	**36.57**			
	Pneumatic hammer	[80510]	Compacting							**78.38**		
	Plate Compactor	[91300]	Compacting					**14.24**	**16.29**		**41.28**	

**Table 6:** Machine + activity identification mode rate comparison between the baseline and novel systems. Relative improvement is calculated as $100\cdot \frac{\left(novel_{accuracy}-baseline_{accuracy}\right)}{baseline_{accuracy}}$.

	Big Excavator			Small Excavator			Pneumatic Hammer	Plate Compactor	Averages
	Moving	Hitting	Scrapping	Moving	Hitting	Scrapping	Compacting	Compacting	
Baseline	49.05%	20.11%	26.03%	50.50%	13.78%	30.22%	71.84%	39.51%	45.15%
Novel	**66.09%**	**22.15%**	**33.74%**	**57.9%**	**14.32%**	**36.57%**	**78.38%**	**41.28%**	**54.92%**
Relative improvement	**34.74%**	**10.14%**	**29.62%**	**12.89%**	**3.92%**	**21.01%**	**9.10%**	**4.48%**	**21.30%**
